# Therapeutic Effects of Medicinal Plants on Cutaneous Wound Healing in Humans: A Systematic Review

**DOI:** 10.1155/2018/7354250

**Published:** 2018-04-01

**Authors:** Tarcisio Vitor Augusto Lordani, Celia Eliane de Lara, Fabiana Borges Padilha Ferreira, Mariana de Souza Terron Monich, Claudinei Mesquita da Silva, Claudia Regina Felicetti Lordani, Fernanda Giacomini Bueno, Jorge Juarez Vieira Teixeira, Maria Valdrinez Campana Lonardoni

**Affiliations:** ^1^Postgraduate Program in Bioscience and Pathophysiology, Universidade Estadual de Maringá, Avenida Colombo 5790, Jardim Universitário, 87020-900 Maringá, PR, Brazil; ^2^Postgraduate Program in Health Sciences, Universidade Estadual de Maringá, Avenida Colombo 5790, Jardim Universitário, 87020-900 Maringá, PR, Brazil; ^3^Department of Clinical Nutrition, Universidade Estadual do Oeste do Paraná, Rua Universitária 1619, Universitário, 85819-110 Cascavel, PR, Brazil; ^4^Medical and Pharmaceutical Science Center, Universidade Estadual do Oeste do Paraná, Rua Universitária 1619, Universitário, 85819-110 Cascavel, PR, Brazil; ^5^Department of Clinical Analysis and Biomedicine, Universidade Estadual de Maringá, Avenida Colombo 5790, Jardim Universitário, 87020-900 Maringá, PR, Brazil

## Abstract

The pharmaceutical industry has made great strides in providing drugs that are able to stimulate the healing process, but only 1–3% of all drugs that are listed in Western pharmacopoeias are intended for use on the skin or cutaneous wounds. Of these, at least one-third are obtained from plants. We sought to review the therapeutic effects of medicinal plants on human skin lesions. For this systematic review, we searched the PubMed, Scopus, and Web of Science databases to identify clinical trials that were published from 1997 to 2017. We reviewed studies that described the use of medicinal plants for the treatment of skin lesions in humans. Ten studies were selected, eight of which were published from 2007 to 2016, with a total of 503 patients. Among the plant species that were used for the treatment of human skin lesions, 12 belonged to 11 families and were included in the analysis. All of the plant species that were studied presented high therapeutic potential for the treatment of cutaneous lesions.

## 1. Introduction

Wounds are physical injuries that result in the opening or rupture of skin, which can cause anatomical and functional disorders. Skin wounds result in the loss of continuity of the epithelium with or without the loss of underlying connective tissue [1]. Causal factors, signaling inhibitors, preexisting conditions, and the type of injury cumulatively determine whether the healing process will be acute or chronic [2]. Wound healing is a dynamic, complex process that leads to the reestablishment of tissue integrity and homeostasis [3] and involves inflammation, reepithelization, granulated tissue formation, neovascularization, wound contraction, and remodeling of the extracellular matrix [4]. This process is coordinated by a complicated signaling mechanism that involves various growth factors, cytokines, and chemokines. Cell proliferation is a necessary step in tissue repair and regeneration during the wound healing process [5]. These injuries constitute a serious public health problem. In the United States, such cutaneous injuries affect thousands of patients and cost billions of dollars to treat [6]. In Brazil, they are also a serious public health problem, although medical records of such injuries are scarce [7].

Wounds that present impaired healing, including acute wounds and chronic wounds, usually do not follow normal physiological healing processes. Such wounds often enter a state of pathological inflammation because of a delayed, incomplete, or uncoordinated healing process [8]. Most chronic wounds are ulcers that are associated with circulatory diseases and diabetes mellitus. Nearly 6 million people suffer from chronic wounds worldwide [9]. In clinical practice, wound dressings and topical products are used to create and maintain a moist environment and provide adequate conditions for healing [10]. However, they are often expensive or ineffective and may generate adverse reactions [11]. Although various pharmaceutical preparations are available, new therapeutic options with fewer adverse effects, a lower cost, and a shorter healing time are continually needed for clinical treatment.

For centuries, plants have been used in both traditional and popular medicine to treat and prevent diseases. In India, “Ayurveda” has been practiced for more than 5000 years as a natural treatment system to prevent and cure diseases, and plants are a part of this process. Traditional Chinese medicine, which is used throughout eastern Asia, is at least 3000 years old and employs numerous plant species [12].

Animal studies of various plant species have generated promising results. For example, *Leea macrophylla* has healing effects. It increases the synthesis of collagen, stimulates the production of antioxidants, reduces the levels of proinflammatory factors, and improves cell proliferation [13]. *Wrightia tinctoria* presented healing activity, with an increase in the rate of contraction of induced lesions [14]. *Pereskia aculeata* accelerated the cicatricial process by increasing blood flow and collagen deposition [10]. An ointment from *Struthanthus vulgaris* stimulated the closure of lesions, stimulated the formation of granulated tissue, and stimulated the proliferation and organization of collagen fibers [15]. *Cynodon dactylon* presented antioxidative activity and stimulated collagen formation and healing [16]. *Caesalpinia mimosoides* stimulated reepithelialization of the epidermal layer and the contraction of lesions [17].

In modern science, plant species that are traditionally used to cure diseases have been extensively studied to identify their bioactive constituents and develop new drugs. Studies of the mechanisms of action and efficacy of these plant compounds have shown that many are pharmacologically safe, warranting further tests in preclinical studies and clinical trials. However, a significant proportion of the world's population cannot afford these modern medicines, and the use of herbal remedies can benefit these patient groups [18].

Considering the importance of the effects of plants and their components on cutaneous tissue, we performed a systematic review to explore the therapeutic effects of medicinal plants on the process of healing cutaneous lesions in humans.

## 2. Materials and Methods

**Figure 1:**
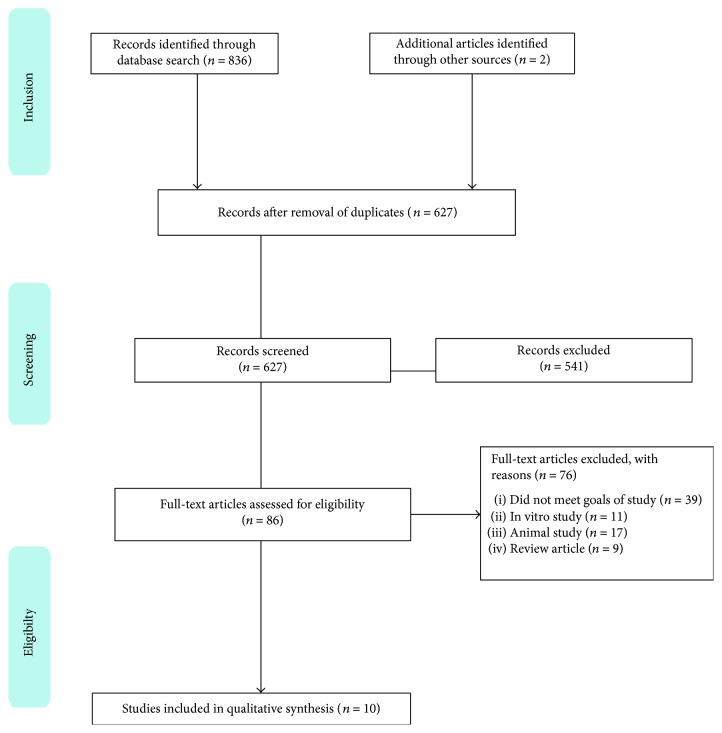
Study flow chart (PRISMA).

This review was conducted in accordance with the recommendations outlined in the Preferred Reporting Items for Systematic Reviews and Meta-Analyses (PRISMA) statement [19]. The search strategy is described in Figure 1.

### 2.1. Search Strategy

A systematic review of the literature was performed using the PubMed, Scopus, and Web of Science databases to search for articles that were published from January 1, 1997, to August 31, 2017. This research was structured according to the characteristics of each electronic database. For the retrieval of publications in PubMed and Scopus, Medical Subject Headings (MeSH) terms were used. Four independent researchers (group 1: TVAL, MT, FBF, and CEL) conducted a specific search to define the maximum MeSH terms that were related to the research goal. Discrepancies were resolved by consensus among the researchers at several meetings. The researchers then presented the MeSH terms to two specialists (JJVT and MVLC) for validation. In the first phase of the study, four researchers performed the search of the databases and analyzed the titles and abstracts. In PubMed, the MeSH terms were organized into three groups: group 1 (Wound healing OR Re-epithelialization OR Regeneration), group 2 (Medicinal plants OR Phytotherapy OR Plant extract), and group 3 (Cytokines OR Collagen OR Fibroblasts OR Inflammation). The blocks were combined separately to allow the largest number of publications (Block 1 AND Block 2 AND Block 3). To identify publications in the Web of Science database, we searched by topics according to the same three-group structure as in PubMed, which guaranteed the identification of studies with high sensitivity.

### 2.2. Study Selection

#### 2.2.1. Inclusion Criteria

Publications that described the use of medicinal plants for wound healing in humans were included in the systematic review. We considered original studies that were published in English, Spanish, or Portuguese within the predefined period of time and for which abstracts were available in the database.

#### 2.2.2. Exclusion Criteria

Review studies, comparison studies, editor's comments, letters, interviews, guidelines, errata, and articles that did not meet the inclusion criteria were excluded.

### 2.3. Quality Assessment

For the second phase of the study, based on publications that were previously defined by the researchers in group 1, the full-text articles were retrieved in PDF format. The articles were randomly and proportionally distributed to the researchers. This step was necessary to determine whether the publications would be retained in the systematic review. Disagreements among researchers were resolved by consensus. In the third phase, the selected papers were randomized and distributed to four independent judges (group 2: MVCL, CMS, CRF, and FGB). This phase is considered highly important because it allows the validation of articles that were selected by the researchers in group 1 in accordance with the inclusion criteria. To increase the sensitivity of this systematic review, we also evaluated the references of the original articles to identify other publications of interest that were not retrieved in the previous database search phases.

### 2.4. Data Extraction

The fourth phase of the research involved data extraction. The four researchers from group 1 with the support of two specialists (JVT and MVLC) organized the structure of the topics to compile the main findings of the publications. The following data are presented in Table 1: reference, year, country, study type, sample, plant family, scientific name, popular name, route of administration, and treatment outcome. The articles were again randomly assigned to the researchers in group 1 to maintain the completeness of the results. The researchers in group 1 then provided the results to the researchers in group 2 for validation. Any discrepancies were resolved by consensus.

## 3. Results

### 3.1. Literature Review

A total of 838 abstracts/citations were identified for preliminary review from electronic and manual searches. The primary search identified 836 articles, with 276 from PubMed, 270 from Scopus, 290 from Web of Science, and two from manual searches of the bibliographies. After removal of duplicates and screening for relevant titles and abstracts, a total of 86 articles underwent a full-text review. Ten articles met the inclusion and exclusion criteria. A flow chart that illustrates the study selection and number of articles at each stage is shown in Figure 1.

### 3.2. Study Characteristics

A total of 503 subjects were evaluated in the 10 studies. The countries where the studies were conducted included the United States (20%, *n* = 2), China (20%, *n* = 2), Brazil (20%, *n* = 2), Mexico (20%, *n* = 2), France (10%, *n* = 1), and Iran (10%, *n* = 1). Eighty percent (*n* = 8) of the articles were clinical trials. The studies were published between 2007 and 2016, and most (30%, *n* = 3) were published in 2011. Regarding the route of administration and treatment mode of the products that were used for wound healing in the selected studies, the topical route of administration was the most commonly used (80%, *n* = 8), followed by intravenous (10%, *n* = 1) and orogastric (10%, *n* = 1; Table 1).

## 4. Discussion

A notable finding was the scarcity of studies that evaluated the healing effects of medicinal plants in humans in the last decade. The few published studies reported important results for the use of medicinal plants, considering their high therapeutic efficacy [20–29].

Recent years have seen the exponential growth of the use of herbal remedies. These agents are gaining popularity in both developed and developing countries because of their relatively low cost, natural origin, and fewer adverse effects [30]. Numerous drugs that are now used in conventional medicine were originally derived from plants [31]. The present systematic review identified 12 plants with healing actions. Among these plants, *Aloe vera*, which is native to Brazil, has been used for thousands of years in folk medicine for the treatment of various diseases.

### 4.1. *Aloe vera*

The *A. vera* plant has proven pharmacological actions, including healing properties, protection of the skin, and anti-inflammatory and regenerative effects [32]. *A. vera* has been used for therapeutic purposes by several cultures because of its cicatrizing effects [33]. This plant has a heterogeneous composition. Its gel contains more than 75 bioactive compounds [34], such as aloe and aloe-emodin (which are responsible for its anti-inflammatory action) and glucomannan (which influences fibroblast growth factor, stimulating its proliferation) [35]. Extracts of *A. vera* stimulate the proliferation of various cell types.

Studies have shown that treatment with *A. vera* gel accelerated the lesion healing process [36, 37]. Increases in wound contraction and collagen synthesis were reported in a 2006 study [38]. *A. vera* also promoted fibroblast proliferation and was shown to play an important role in remodeling the extracellular matrix during healing [39].

*A. vera* and collagen were topically applied for the treatment of an ischemic lesion in a patient with arterial hypertension and diabetes mellitus. At the end of approximately 10 weeks of treatment, the lesion completely healed. The topical application of *A. vera* provides more oxygen, increases vascularity, and increases collagen production, thus promoting wound healing. It also promotes the multiplication of epithelial cells and remodels and heals the lesion [40].

### 4.2. *Salvia miltiorrhiza*

*S. miltiorrhiza* (*Salvia*) has an Asian origin. It is widely used in traditional Chinese medicine to treat various cardiovascular and cerebrovascular diseases, hyperlipidemia, tumors, and liver problems, such as cirrhosis. It may also influence the recovery of damaged tissue [41]. More than 70 compounds were identified in *Salvia*, and many of these bioactivities were attributed to two main groups of compounds: salvianolic acids and tanshinones [42, 43]. The tanshinone group has been extensively studied and reported to have several pharmacological actions, such as anti-inflammatory and antioxidant effects [44].

*S. miltiorrhiza* improves the healing process by removing necrotic cells, attenuating the inflammatory response, decreasing apoptosis [45, 46], reducing oxidative stress [45], and possibly facilitating the repair of tubular epithelial structures. A rat study evaluated a transgenic *S. miltiorrhiza* extract that was applied topically to burn wounds, which increased fibroblast growth factor 1 activity 19.2-fold, increased cell proliferation, accelerated the growth of new blood vessels, and reduced healing time [47]. These results corroborate those of Chen et al. [21].

### 4.3. *Mimosa tenuiflora*

*M. tenuiflora* is a leguminous tree that is found in the semiarid region of northern Brazil. It is used medicinally for its curative properties. The stem bark and roots of this tree are widely used by indigenous tribes for psychoactive and medicinal purposes [48]. *Mimosa*'s potential to heal severe skin ulcers was attributed to its high polyphenol content [49] and another group of triterpenic saponins in its bark, mimonosides A-C [50], which induce the proliferation of human cells in culture and have immunomodulatory effects [51]. A *Mimosa* bark extract was used to treat varicose ulcers and had antimicrobial actions in vitro against a large group of microorganisms [24]. In association with *M. tenuiflora*, the effects of *Alchemilla vulgaris* were evaluated by Shrivastava [22] in a clinical trial.

### 4.4. *Alchemilla vulgaris*

*A. vulgaris* originated in northern Europe and the mountainous regions of southern Europe. It is commonly known for its astringent and anti-inflammatory properties. It is traditionally used to treat ulcers, eczema, and dermatitis. Similar to other members of the Rosaceae family, *A. vulgaris* contains polyphenols that are responsible for the major pharmacological actions of the plant. Its extract is rich in proanthocyanidins, which are multiple polymers that are composed of anthocyanidin [52] and have an affinity for proteins [53, 54]. Procyanidins have been shown to have a strong affinity for proteolytic enzymes, such as elastase, xanthine oxidase, *β*-glucuronidase, collagenase, and hyaluronidase, which are involved in the destruction of cell matrix components [52, 55].

Potential wound healing properties have been described for the major components of *A. vulgaris* [56, 57]. The healing effects of this plant on cutaneous lesions were tested in rats. The results showed healing properties that were associated with the promitotic activity of epithelial cells and myofibroblasts [58].

### 4.5. *Angelica sinensis*

*A. sinensis* originated in Asia and has been used in traditional Chinese medicine for more than 2000 years, mainly to treat gynecological problems, anemia, and wounds [59, 60]. An *Angelica* extract induced the cicatrization process, which appeared to occur through multiple actions, such as inhibition of the production of reactive oxygen species (ROS), an increase in cell mobility, the promotion of glycolysis, the inhibition of apoptosis, and an increase in the proliferation of fibroblasts [61]. An in vivo study evaluated an SBD.4 isolate from *Angelica* and found significant increases in healing strength, tissue regeneration, and type I collagen [23]. *A. sinensis* polysaccharide was shown to have a healing effect on experimental gastric ulcers and an in vitro stimulating effect on the proliferation of gastric epithelial cells [62].

### 4.6. *Origanum vulgare* L.

*O. vulgare* L., commonly known as oregano, is native to the entire Mediterranean region, Euro-Siberian region, and Irano-Turanian region. Its essential oils have been used since ancient times to relieve various ailments, such as coughs, colds, skin conditions, and digestive disorders [63, 64]. The main constituents of the extract of this plant include hydrocarbons, monoterpenes, and phenolic compounds. Carvacrol and thymol are the major products of this plant and are responsible for its antioxidant and antibacterial actions [65]. It also has potential anti-inflammatory effects by inhibiting lipoxygenase and acetylcholinesterase [66].

A rich essential oil of this plant consists of monoterpenes and sesquiterpenes (e.g., caryophyllene and spatulenol), which have antioxidant, antifungal, and antimicrobial effects against various types of bacteria, fungi, and yeast [67, 68]. Another bioactive constituent of *O. vulgare* is protocatechuic acid, which has high antioxidant capacity and is able to eliminate free radicals by inhibiting lipid peroxidation and suppressing ROS [69]. An in vivo study of titanium dioxide nanoparticles that contained a leaf extract of *O. vulgare* reported significant skin healing activity [70].

### 4.7. *Lavandula stoechas* L.

*L. stoechas* L. is a small aromatic shrub that is found in southwestern Europe, the Middle East, and North Africa. It is widely used in traditional medicine to treat various diseases. The essential oil of lavender has beneficial effects on wound healing [71–73]. Clinical trials have suggested a beneficial effect of lavender oil on wound healing. Topical treatment with lavender oil significantly reduced the size of foot-and-mouth ulcerations compared with controls [72]. In another study, wound closure progressed more rapidly with topical application of lavender oil compared with controls, accompanied by an increase in the expression of platelet-derived growth factor-A and epidermal growth factor, thus demonstrating its beneficial effects on tissue remodeling and reepithelialization [73].

*L. stoechas* L. essential oil has antioxidant, broad-spectrum antimicrobial, antifungal, and anti-inflammatory actions [74]. Algieri et al. reported the anti-inflammatory effect of a hydroalcoholic extract of *L. stoechas* L. in both in vitro and in vivo assays [75]. The effects of a 10% extract of *L. stoechas* L. on open lesions were evaluated in rats, with greater lesion contraction and significant histological results compared with controls [76].

*L. stoechas* L. oil had positive effects on wound healing in rats and was recommended for the treatment of chronically infected wounds because of its immunostimulating action and antimicrobial effects [77]. However, to our knowledge, only one study has evaluated its healing effects in humans [26].

### 4.8. *Radix astragali*

*R. astragali* is a well-known herb in traditional Chinese medicine. The main constituents of *R. astragali* include polysaccharides, saponins, flavonoids, amino acids, and trace elements [78]. A saponin- and isoflavone-enriched extract of *R. astragali* promoted angiogenesis in human endothelial cells [79], and calicossin is known as an angiogenesis-promoting isoflavone [80]. Recent studies have shown that flavonoids in *R. astragali* have strong antioxidant activity and pharmacological effects, such as mitigation of the deleterious effects of hypoxia [81] and anti-inflammatory, antioxidative, immunoregulatory, and neuroprotective actions [82].

A randomized, double-blind, placebo-controlled study of two herbal formulations found that they effectively promoted the healing of chronic diabetic ulcers, with recovery in 85% of patients [83]. Among the herbal components, *R. astragali* and *Rehmanniae radix* were shown to have the most pronounced effects. These two species were shown to stimulate the growth of fibroblasts in diabetic foot ulcer tissue in diabetic patients [84, 85].

### 4.9. *Rehmanniae radix*

*R. radix* is classified as a very effective herb in traditional Chinese medicine. It consists of approximately 70 monomers. Polysaccharides, oligosaccharides, stachyose, and monosaccharides have been identified in *R. radix* in high concentrations [86]. Recent studies have shown that *R. radix* and its active constituents have a wide spectrum of pharmacological actions in the circulatory, immune, endocrine, cardiovascular, and nervous systems [87]. Its aqueous extract stimulated the proliferation of fibroblasts and effectively healed diabetic foot ulcers in rats through tissue regeneration, angiogenesis, and inflammation control processes [84, 85].

### 4.10. *Ageratina pichinchensis*

*A. pichinchensis* is a member of the Asteraceae family. It has been used for many years for the treatment of cutaneous lesions. Preliminary pharmacological tests of extracts of *A. pichinchensis* revealed a shorter time of cicatrization of surgically induced cutaneous wounds in rats [88]. One study identified and isolated the flavonoid 7-*O*-(*β*-d-glucopyranosyl)-galactin, to which the cell proliferation capacity of human skin was attributed [89].

A double-blind, controlled clinical study evaluated the effects of topical application of a pharmaceutical formulation that contained an extract of *A. pichinchensis* in patients with chronic venous leg ulcers, and 100% therapeutic efficacy was found [28]. Antimicrobial and anti-inflammatory effects [89] of extracts of this species have also been described, which may contribute to its wound healing effects.

### 4.11. *Calendula officinalis*

The most recent study that was identified in the present systematic review evaluated *C. officinalis* L. This plant has been cultivated in Europe for centuries and is used medicinally throughout the world [90]. The main constituents of *C. officinalis* are pentacyclic triterpenes, including *ψ*-taraxastene, taraxastene, lupene, Δ12-oleanene, and Δ12-ursene [91]. These triterpenes exist as monols, diols, and triols in free form, esterified with long fatty acids or as oleanene-type saponins. Triterpenes in *C. officinalis* flowers can balance the inflammatory response and may be beneficial for the treatment of chronic wounds that are associated with chronic inflammation [92]. A 5% marigold tincture was shown to have cicatrization actions [93], with an increase in cells that are involved in the cicatricial process, resulting in a more satisfactory healing response in rabbits, compared with other treatments that were applied to experimental skin wounds. *C. officinalis* treatment in patients with venous ulcers accelerated healing [94]. Leach [95] performed a systematic review of several clinical trials and concluded that *C. officinalis* has several properties that are conducive to wound healing.

Medicinal plants are important alternatives for the healing of lesions. Their use has been validated in clinical trials that evaluated their beneficial and adverse effects. Further studies are needed to demonstrate their clinical efficacy and safety and evaluate costs and benefits.

One limitation of the present study was that we searched only three databases. Another limitation was that we only evaluated studies that were published in English, Spanish, and Portuguese. Furthermore, we only included papers that had summaries/abstracts available online.

## 5. Conclusions

A total of 12 medicinal plants and herbal compounds have been studied in clinical trials and case studies with regard to their wound healing actions. Only one plant species was used in more than one publication (by different authors). These 12 plants were reported to have high skin lesion healing potential, with significant differences compared with those of controls. Two case studies described the complete healing of lesions in patients. *Mimosa tenuiflora* was the most studied plant in the last decade. Two studies reported its potential wound healing properties, with 100% efficacy for the treatment of lesions.

## Figures and Tables

**Table 1:** Characteristics of included studies.

Reference	Year Country	Study type	Sample	Scientific name (plant family)	Popular name in Brazil	Route of administration/treatment mode	Outcome
[20]	2010 Brazil	Case report	1	*Aloe vera* (Liliaceae)	Babosa	The dressing was composed of sterile gauze for topical use, with an extract of *A. vera* in gel (1.5 ml), collagen (2.0 g), glycerin (5.3 ml), and paraben (0.1 g) preservatives included in its composition.	Treatment with the product ceased on August 22, for a total treatment duration of 2 months 11 days. The healing process was complete.
[21]	2010 China	Clinical trial randomized	90	*Salvia miltiorrhiza* (Lamiaceae)	Sálvia	20 ml intravenously every 12 h from the day of surgery until 3 days after surgery.	At 4 days after surgery, the women in the control group had significantly more ischemia and necrosis compared with group 2 (*Salvia miltiorrhiza*, *p* = 0.002) and group 3 (anisodamine, *p* < 0.001). No significant difference was found between groups 2 and 3 on postoperative day 4 (*p* = 0.125). Similarly, on day 8, ischemia and necrosis in group 1 (control) were significantly more severe than in groups 2 and 3 (both *p* < 0.001).
[22]	2011 France	Clinical study	101	*Mimosa tenuiflora* (Leguminosae)	Jurema-preta	The test product tube was opened, and the product was applied drop by drop on the boundary of the wound such that the product formed a thin film over the entire wound surface. The wound was then covered with sterile cotton gauze to allow some air circulation. The wound was examined twice daily and cleaned if necessary, and fresh product was applied in an identical fashion.	The reduction of the mean wound surface area was much faster in the AS-21-treated group (reduction from 52.03 ± 36.66 cm^2^ on day 0 to 16.7 ± 20.5 cm^2^ [67.90%] on day 21 and 2.13 ± 3.86 cm^2^ [97.87%] on day 42). Complete healing was observed in 19 of 69 wounds (27.53%) after 4 weeks and in 41 of 69 wounds (59.4%) after 6 weeks. All of these results were statistically significant compared with the corresponding placebo values from day 14 onward (*p* < 0.005).
				*Alchemilla vulgaris* (Rosaceae)	Pé de leão; alquemila		
[23]	2012 United States	Clinical case studies	4	*Angelica sinensis* (Apiaceae)	Angélica Chinesa	Sterile SBD.4 dressings were applied on lower-extremity chronic ulcers.	All wounds healed when the experimental SBD.4 dressing was applied.
[24]	2007 Mexico	Clinical trial	40	*Mimosa tenuiflora* (Leguminosae)	Jurema-preta	Treatment consisted of once-daily washings of the ulcerated area with clean boiled water and neutral soap followed by application of the hydrogel. The lesion was then covered with a simple dressing and compression bandage.	With this experimental treatment, 57.89% of the patients presented therapeutic efficacy at the end of week 4 of treatment, and nearly 100% presented therapeutic efficacy at the end of week 8. In the placebo group, only one patient presented therapeutic efficacy at week 6 (*p* = 0.0001).
[25]	2011 United States	Clinical trial	40	*Origanum vulgare* (Labiatae)	Orégano	The study ointment was applied to the excision site by a medical assistant. The evaluating physicians were blinded to treatment. The excision site was then covered with a nonoclusive dressing. On day 0, the study ointment was provided to the patient in a concealed container, and the patients were instructed to apply the ointment twice daily to the site and cover it with a nonoclusive dressing.	The oregano ointment group had lower scar assessment scores (i.e., closer to normal skin) compared with the petrolatum group with regard to color, stiffness, thickness, and irregularity. The oregano ointment group presented statistically significant improvement in color compared with the petrolatum group on day 12 (*p* = 0.04) and day 45 (*p* = 0.009). Scar assessment by the physicians indicated that the oregano ointment group had lower scar scores, with closer to normal skin, than the petrolatum group in all five categories: pigmentation, vascularity, thickness, relief, and pliability. In the physician scar assessment, the oregano ointment group presented statistically significant improvement in pigmentation compared with petrolatum on day 12 (*p* = 0.0014) and significant improvement in pliability on day 90 (*p* = 0.05).
[26]	2011 Iran	Clinical trial	120	*Lavandula stoechas* (Lamiaceae)	Rosmarinho	Controls and cases received povidone-iodine and essential lavender oil, respectively. A sitz bath using 5–7 drops of essential lavender oil in 4 l of water, twice daily for 10 days, was used in the case group. The control group received routine postnatal care using povidone-iodine.	A total of 25 subjects in the lavender group and 17 in the control group reported no pain at all, with no significant differences between groups. Thirty-one subjects (51.7%) in the lavender group and 13 subjects (21.7%) in the control group had no redness (*p* = 0.001). Edema > 2 cm was not observed in the lavender group. No complications were observed, with the exception of mild irritation in two patients. Three cases and two controls had mild infections that were treated by antibiotics.
[27]	2015 China	Clinical trial	16	*Radix astragali* (Leguminosae)	Astragalus	An NF3 powder that contained extract granules was formulated into sachets, and the subjects were instructed to take two sachets daily (5 g/sachet) for 6 months.	At the end of study, the wound areas decreased in six patients after NF3 treatment compared with only one patient in the placebo group. The mean wound area decreased by 47.8% and 14.1% in the NF3 and placebo groups, respectively (*p* < 0, 01). Patients in the NF3 group presented a steady decrease in wound areas compared with marked fluctuations in the placebo group during clinic visits. The mean healing time was 125 and 137 days in the NF3 and placebo groups, respectively.
				*Rehmanniae radix* (Scrophulariaceae)	Rehmannia		
[28]	2012 Mexico	Clinical trial	34	*Ageratina pichinchensis* (Asteraceae)	Bustamenta	The experimental group received treatment with a standardized (0.76% encecalin) *A. pichinchensis* extract. The control group received 7% propylene glycol alginate. Both treatments were only applied once weekly on the previously cleaned wound area until complete wound healing was achieved over a maximum 10-month period.	Ulcer size diminution was significantly higher (*p* < 0.010) in patients who received the experimental treatment with the *A. pichinchensis* extract. During the first month of treatment, a 49.8% decrease in ulcer size was observed in the experimental group. The control group presented a 19.1% decrease (*p* < 0.01). In the second month of treatment, ulcer size diminution reached 79.1% and 41.6% in the experimental and control groups, respectively (*p* < 0.013). In the third month of treatment, ulcer size diminution was 89.1% and 63.1%, respectively, in the experimental and control groups, respectively (*p* < 0.056). Similar results were observed during the subsequent months of treatment, with significant differences between the experimental and control groups.
[29]	2016 Brazil	Clinical trial	57	*Calendula officinalis* (Asteraceae)	Calêndula	Chronic venous leg ulcers were cleaned twice daily with 25 ml of sterile physiological saline solution immediately before applying an extract of *C. officinalis* or the standard care product. The extract was sprayed on the wound bed and allowed to dry for a few minutes. Conventional dressings with sterile nonadherent gauze and nonelastic supportive bandages were used in all patients. The nursing team instructed patients or their caregivers to apply the wound dressings twice daily after using the specific treatment.	At the end of the 30-week study, 73.7% of the patients who were treated with the extract achieved complete epithelialization, with an average healing time of 13.3 ± 5.9 weeks. The proportion of completely healed patients in the control group was 31.6%, with an average healing time of 22.1 ± 5.9 weeks. The mean wound contraction in patients who were treated with the extract was 42.7 mm^2^/week, which was significantly greater (*p* < 0.001) than that in patients in the standard treatment control group (12.3 mm^2^/week).

## Supplementary Materials

Supplementary Materials Preferred Reporting Items for Systematic Reviews and Meta-Analyses (PRISMA) statement [19].
